# Eight quick tips for using the reactome knowledgebase

**DOI:** 10.1371/journal.pcbi.1014891

**Published:** 2026-10-27

**Authors:** Nancy T. Li, Marija Milacic, Karen Rothfels, Peter D’Eustachio

**Affiliations:** 1 Ontario Institute for Cancer Research, Toronto, Ontario, Canada; 2 NYU Grossman School of Medicine, New York, New York, United States of America; Montreal, CANADA

## Introduction

A central goal of genomics and bioinformatics is to systematically annotate the functions of human proteins, providing standardized descriptions that support computer-based exploration of human biology and disease. Together, these annotations provide a structured representation of the molecular components and processes that underlie human biology. Reactome is a curated knowledgebase that contributes to this effort by systematically annotating the functions of human proteins and representing human molecular physiology as a connected set of biological processes [1]. It can be used as a searchable on-line textbook of human molecular physiology and as a tool for interpretation of high-throughput proteomic data sets. Users can move from individual genes, proteins, or reactions to the biological pathways in which they participate.

To understand whether Reactome is an appropriate tool for your research problem, it is useful first to review the design and implementation of the knowledgebase (Fig 1). The tips below describe how to use these different capabilities, while Box 1 provides additional detail on the data model that underlies the resource. Reactome currently (June 2026) contains annotations of 16,423 reactions involving 11,694 human proteins (of an estimated 20,533 [https://www.ensembl.org/Homo_sapiens/Info/Annotation?redirect=no]) and 2,188 small molecules, backed by data from 43,308 publications. Reactome is actively curated and updated quarterly, so the content and pathway representations available to users change over time.

**Fig 1:**
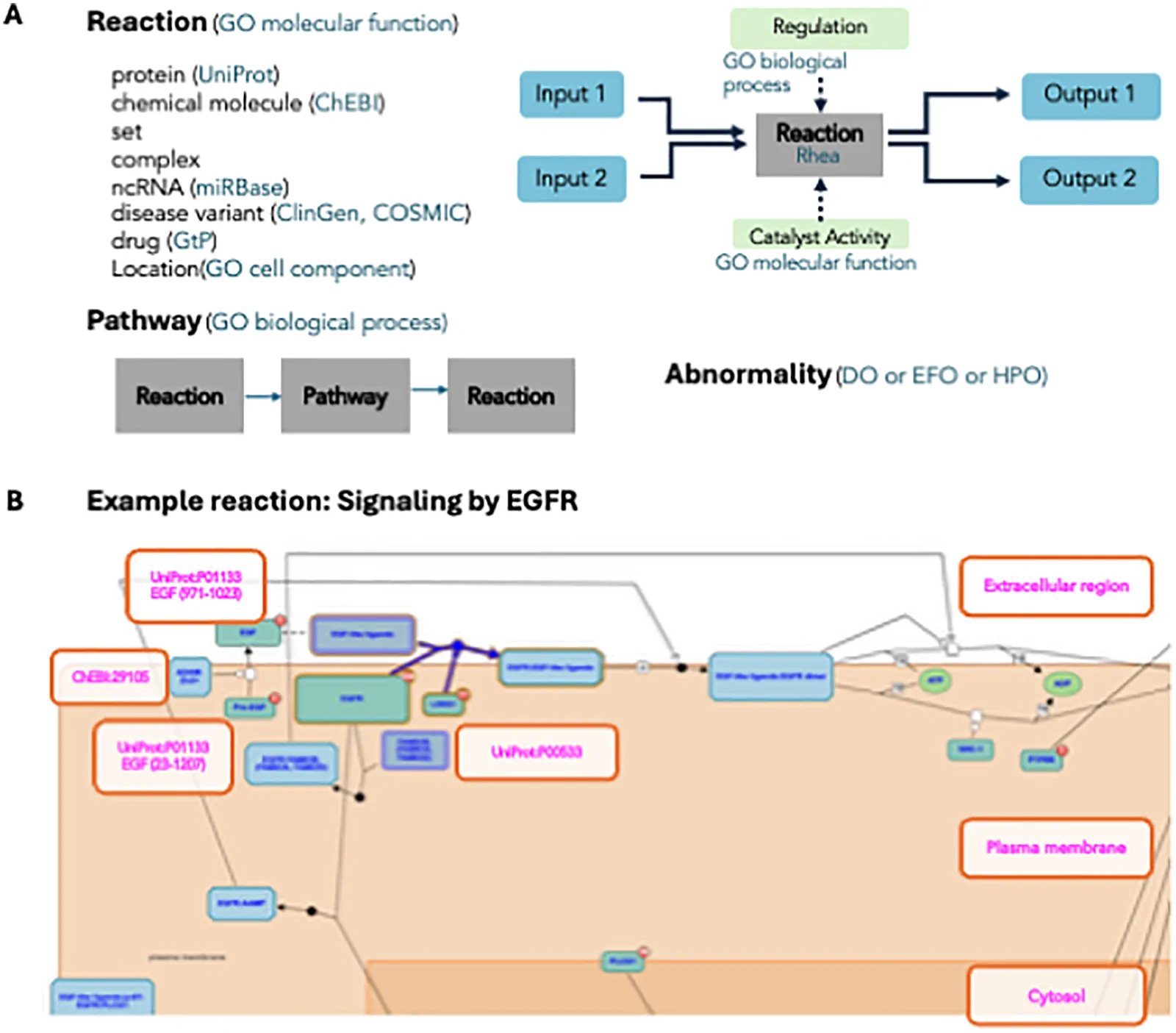
The Reactome data model. **(A)** Diagram of a Reactome reaction (above) and linkage of reactions into causal pathways (below), showing components and their relationships to community data resources. **(B)** Example of a reaction sequence in Reactome (R-HSA-177929). All reactions and their participant entities have a cellular location (compartment) indicated. Pro-EGF, located in the plasma membrane, is cleaved by a metalloprotease to form active extracellular EGF. EGF becomes an input to the next reaction, in which it binds EGFR. This reaction is negatively regulated by LRIG1 and targets EGFR for ubiquitination and degradation. The resulting receptor–ligand complex then becomes an input to the following reaction.

Reactome annotations provide an expert consensus view of the molecular steps that make up a human biological process, identifying the proteins and small molecules involved in each, specifying their interactions, and providing links to key publications that describe the evidence that supports this view with cross-references to relevant data in other resources. Rather than serving as a comprehensive repository of all published observations, Reactome synthesizes evidence into a structured representation of biological processes. Reactome annotations are meant to be terse and current, not comprehensive catalogs of available evidence nor histories of how the current views arose.

Reactome uses community protein and small molecule data resources as single sources of truth to annotate reactions (Box 1). This integration allows specialized resources to contribute to a coherent and up-to-date representation of biological knowledge, and allows Reactome entities to be linked consistently to their canonical representations in external resources. Some Reactome events are inferred from experimentally characterized reactions involving orthologous proteins in other organisms; these inferred events are identified in the pathway hierarchy.

**Box 1: Understanding the Reactome Data Model**

The Reactome data model (Fig 1A) builds on classic metabolic maps [e.g., http://www.iubmb-nicholson.org/index.html], first implemented on-line by KEGG [2] and EcoCyc/MetaCyc [3, 4], extending them to domains of molecular physiology including gene expression, the cell cycle, differentiation and development, immune function, and diseases caused by infectious agents and inherited or somatic mutations.

Reactions are single-step events in which input physical entities are transformed into output ones, enabled by proteins and protein complexes, and positively or negatively regulated by other physical entities. Transformations include covalent reactions of entities, transport of entities between subcellular locations, and assembly and disassembly of multimolecular complexes. Shared physical entities connect reactions, i.e., the output of one is an input, catalyst, or regulator of a following one within or between pathways (Fig 1). This connected representation allows users to move from individual genes, proteins, or reactions to the larger biological processes in which they participate.

Chemical entities in Reactome are annotated as variants of the canonical forms of these entities described by ChEBI [5] and RHEA [6], distinguished from one another by subcellular location. Proteins are annotated as variants of the canonical forms of these entities described by UniProt [7], distinguished from one another by posttranslational modifications (annotated with ChEBI “group” instances), subcellular location, and the cell type in which each entity is found. Gene Ontology terms are used to identify molecular functions of proteins, their roles in multistep processes, and their subcellular locations [8]. Referring the working forms of a physical entity to a canonical form enables users to find all annotations of a canonical form and its variants, and to navigate to the definitive description of that canonical form maintained by ChEBI or UniProt.

In addition to fully annotated single-step reactions, Reactome uses two kinds of partly annotated black box reactions to provide causal connectivity in pathway descriptions and minimize re-annotation. For connectivity, a black box “unknown” event describes a transformation that is known to occur, but that is incompletely characterized. For example, a preprotein is activated by cleavage by an unknown protease. To minimize reannotation, a black box “omitted” event represents a specific instance of a multistep process that has been annotated elsewhere in Reactome. For example, transcription of a generic protein-coding gene to yield its mRNA is a fully annotated pathway. To annotate the expression of a specific gene as part of, e.g., transcriptional regulation of energy metabolism, a black box “omitted” event is created with the gene as input, transcription factors as regulators, and its mRNA as output, which stands in for the full sequence of events needed to transcribe that gene.

The following tips illustrate practical ways to use Reactome for exploring biological processes (Fig 2), analyzing data (Fig 3), and working with the resource (Fig 4).

**Fig 2:**
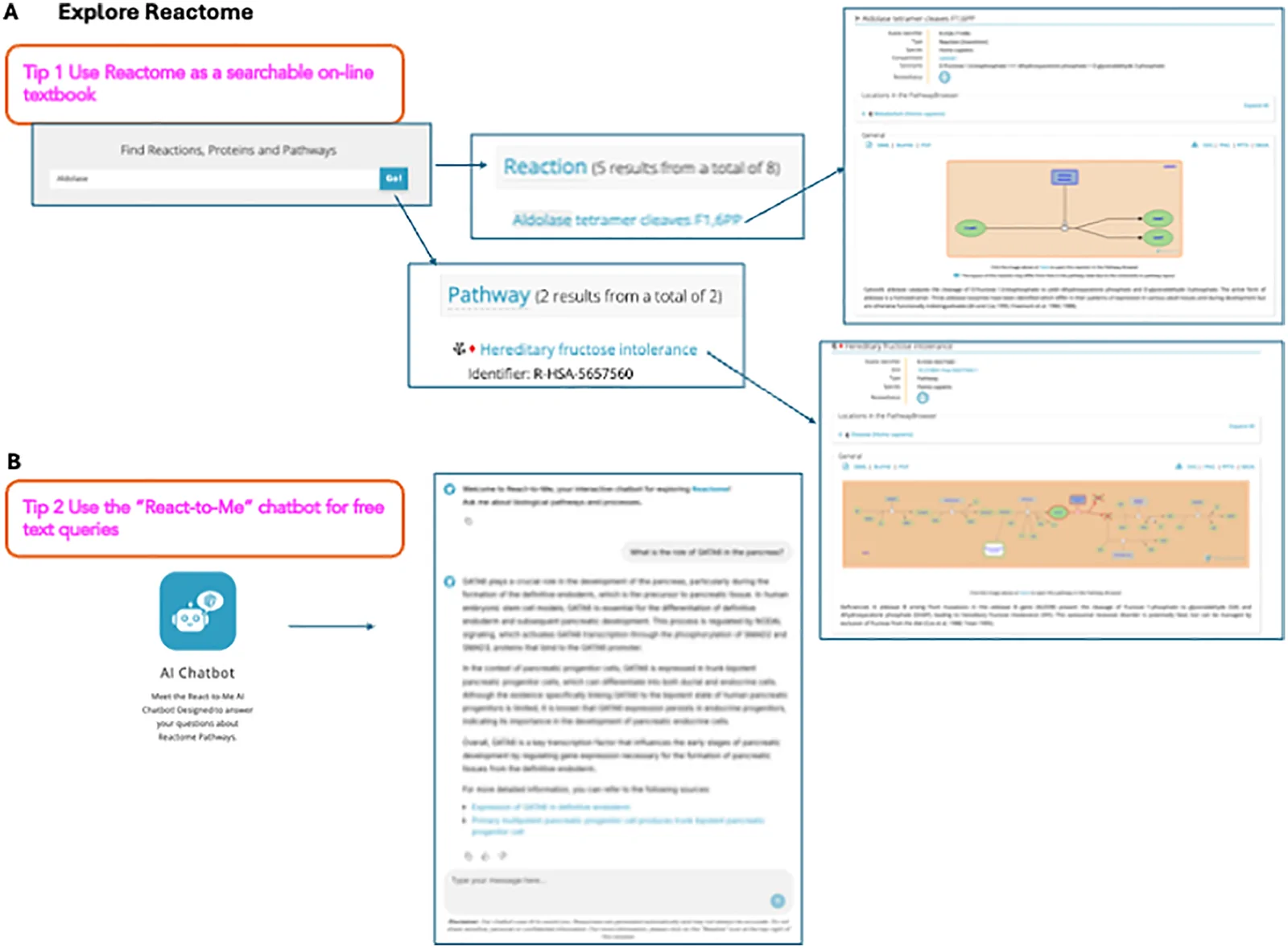
Screen captures showing two ways to explore Reactome, following the examples described in (A) Tip 1 and (B) Tip 2.

**Fig 3:**
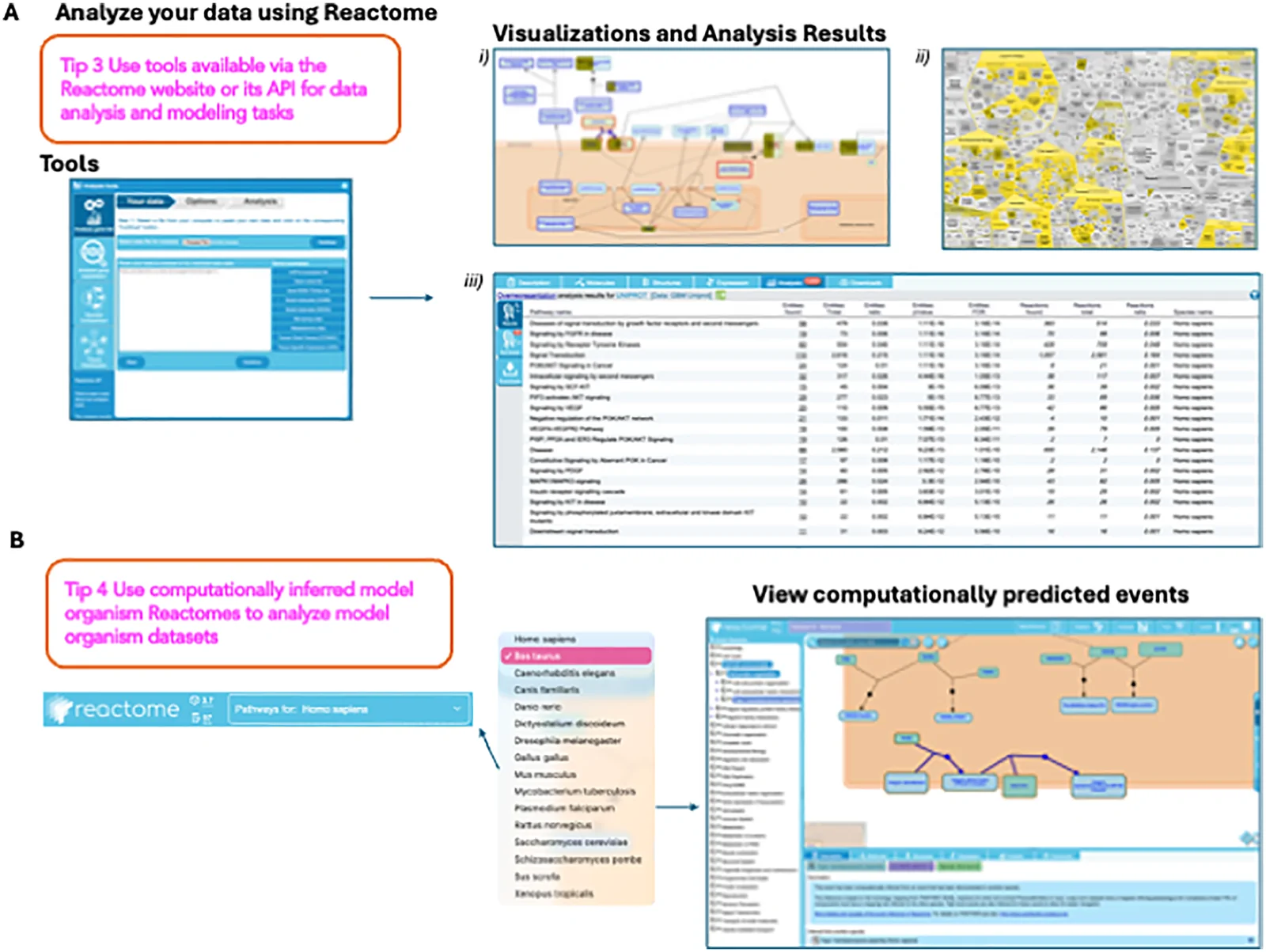
Screen captures showing examples of user workflows for (A) Tip 3, using the Reactome web interface to perform overrepresentation analysis, with (i) molecular overlay, (ii) Voronoi diagram, and (iii) analysis results, and (B) Tip 4, showing computationally inferred events in a non-human organism, in this example *Bos taurus.*

**Fig 4:**
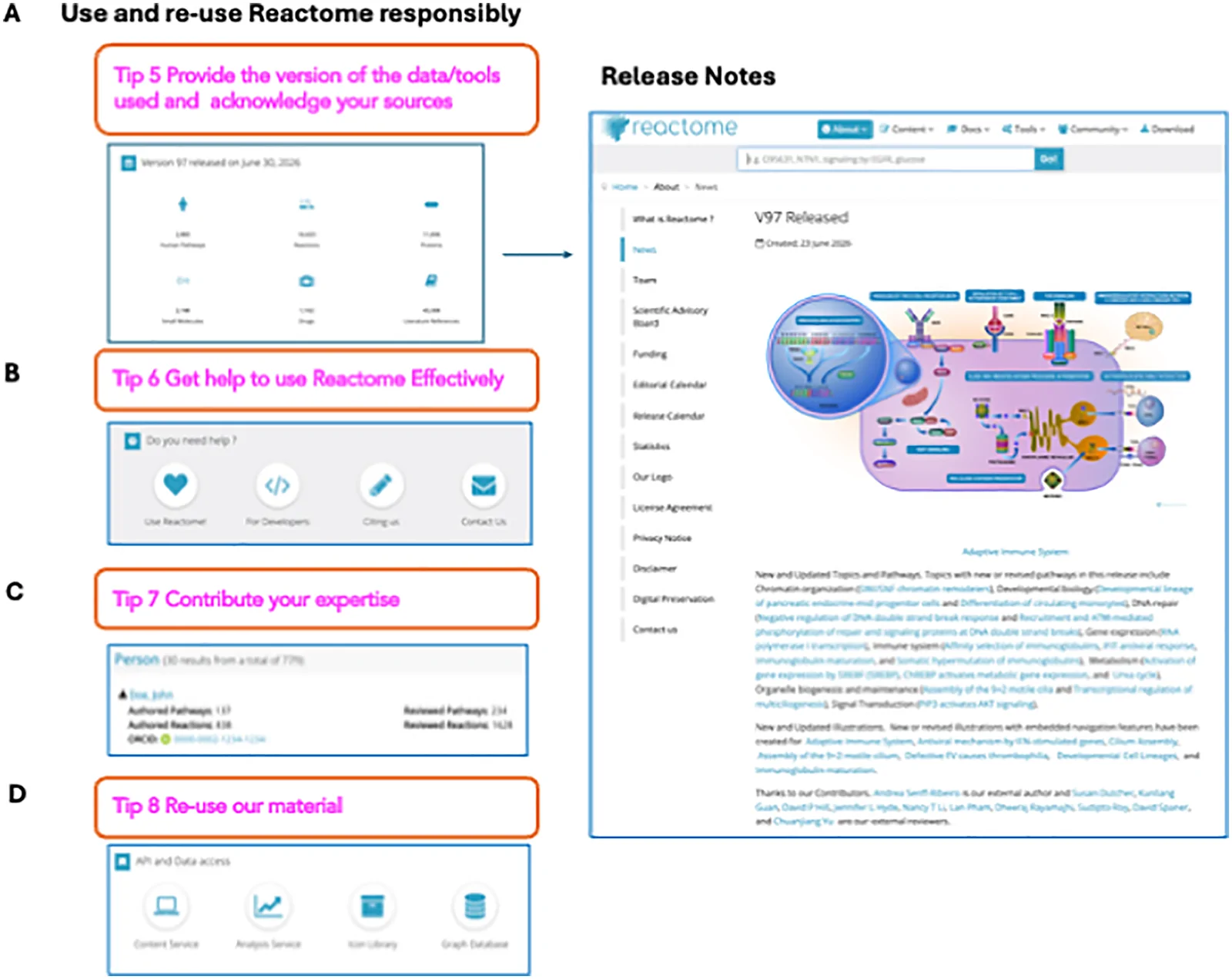
Screen captures showing examples of resources and features described in (A) Tip 5, including the Reactome news section and release notes; (B) Tip 6, including links to user and developer documentation, citation information, and the Reactome helpdesk; (C) Tip 7, showing ORCID attribution for community contributors who author or review pathways; and (D) Tip 8, showing API and data access options available through the Reactome website.

### Tip 1: Use Reactome as a searchable on-line textbook

Use Reactome to explore how genes, proteins, small molecules, and biological processes are connected. The Reactome website provides both a hierarchical view of biological pathways and structured searches for individual entities and events.

Our primary user interface, www.reactome.org, provides access to the repository via a human-facing browsable and searchable interface. Pathways are laid out as a hierarchy of superpathways (e.g., metabolism, DNA replication) that can be unfurled to reveal subpathways, and, ultimately, single-step reactions. When an event at any level of this hierarchy is selected, its details are displayed in a standard human-readable format. A user can navigate, for example, from “Metabolism” to “Carbohydrate metabolism” to “Glucose metabolism” to “Glycolysis” to “Aldolase tetramer cleaves F1,6PP”. Alternatively, a user can search directly for a gene, protein, chemical, or event. For example, searching for “aldolase” yields the reactions and pathways in which this enzyme family participates, as well as related disease processes and inferred events involving model-organism orthologs. (see Tip 4).

### Tip 2: Use the “React-to-Me” chatbot for free text queries

Use the React-to-Me chatbot, https://reactome.org/chat/, when your question is broader than a single gene, protein, or pathway, or when you are not sure which Reactome terms to search for. The chatbot accepts questions in English and over 20 non-English languages, and retrieves relevant curated content from Reactome, allowing users to explore connections across multiple pathways.

For example, a question such as “What is the role of GATA6 in the pancreas?” requires information from several areas of Reactome rather than a single pathway. React-to-Me can identify relevant curated content and present the results as a combination of text, pathway diagrams, illustrations, and links to related resources.

More information about the chatbot can be found in our preprint [9].

### Tip 3: Use tools available via the Reactome website or its application programming interface (API), for data analysis and modeling tasks

Use Reactome’s pathway analysis tools and specialized tools for interpreting high-throughput datasets in the context of biological pathways. Depending on the type of data and question, users can perform basic bioinformatics workflows such as qualitative and quantitative gene set enrichment and cross-species and cross-tissue pathways comparison (Table 1) [10–12]. Results can then be explored in the context of the underlying Reactome pathway hierarchy and reactions.

**Table 1:** A summary of Reactome features for data browsing and data analysis.

Category	Tool/Resource	Mode of Access	Primary Purpose	Typical Input Data	Typical Use Case
Interactive Web Applications (Exploration & Visualization)	Reactome Pathway Browser https://reactome.org/PathwayBrowser/	Web	Interactive pathway exploration and visualization	Gene/protein/metabolite identifiers	Browse pathway context, inspect molecular events, visualize pathway hierarchy
	Reactome Expression Analysis https://reactome.org/PathwayBrowser/#TOOL=AT	Web	Overlay quantitative expression/abundance data on pathways	RNA-seq, proteomics	Visualize pathway activity changes across conditions
	Reactome Species Comparison https://reactome.org/PathwayBrowser/#TOOL=AT	Web	Cross-species pathway comparison using orthology	Gene/protein/metabolite identifiers from model organisms	Compare conservation of pathways between human and model species
	Reactome Tissue Distribution https://reactome.org/PathwayBrowser/#TOOL=AT	Web	Visualize tissue-specific distribution of pathway components using expression data	Gene/protein identifiers, pathway components	Assess tissue-specific expression of pathway-associated molecules
	ReactomeIDG https://idg.reactome.org/	Web	Predict interacting pathways for understudied or dark proteins that would not be contained in the curated Reactome Knowledgebase	Gene/protein identifier, typically understudied gene/protein	Study dark proteins in the context of Reactome pathways and learn potential functions of these proteins
Programmatic Analysis Services (APIs & Computational Access)	Analysis Service API https://reactome.org/AnalysisService/	API	Automated pathway enrichment analysis	Omics feature lists	High-throughput pathway enrichment workflows
	Content Service API https://reactome.org/ContentService/	API	Retrieve pathway hierarchy and detailed annotations	Programmatic requests	Query pathway metadata and molecular events
	ReactomeGSA https://reactome.org/gsa/home	Web/R package	Comparative multi-omics pathway analysis across datasets	User or Public Transcriptomics, proteomics, RNA-seq data	Gene set enrichment analysis and cross-study pathway comparisons
Workflow Integration Platforms	Reactome Galaxy Integration https://github.com/reactome/reactome_galaxy	Galaxy platform	Incorporate Reactome analysis into reproducible workflows	Gene lists, omics matrices	Automated enrichment analysis in multi-step pipelines
	ReactomeFIViz https://reactome.org/tools/reactome-fiviz	Cytoscape plugin	Functional interaction network construction and analysis	Gene lists, mutation data, expression matrices	Network analysis, clustering, module discovery
Data Export & Knowledge Resources	Reactome Graph Database https://reactome.org/dev/graph-database	Download	Graph-based querying of biological relationships	Graph queries	Network traversal and mechanistic inference
	BioPAX Export https://reactome.org/download-data	Download	Export pathways in standardized exchange format	Pathway data	Systems biology integration and network modeling
	SBML Export https://reactome.org/download-data	Download	Export pathways for mathematical modeling	Pathway models	Dynamic simulation and ODE-based modeling
	Reactome Content Download https://gallery.ecr.aws/reactome/graphdb	Download	Bulk access to complete curated knowledgebase	Full database dump	Large-scale computational analysis, ML, knowledge graphs

Reactome also provides tools for more specialized analyses. Analysis tools can be used to interpret high-throughput omic-scale data and provide the framework for building mathematical models of biological processes. ReactomeFIViz [13] and ReactomeIDG [14] provide additional capabilities for analyzing interaction and perturbation effects among genes of interest, based on their annotated Reactome roles and coregulation data imported from external sources. The appropriate tool depends on the type of data available and the intended biological question, as summarized in Table 1. An example is also described in Fig 3.

### Tip 4. Use computationally inferred model organism Reactomes to analyze model organism data sets

For researchers analyzing non-human organisms, Reactome offers computationally inferred pathway representations for 14 model organisms. Inference based on sequence similarity is used to computationally infer pathway networks for each of 14 model organisms. If a model organism has orthologs of the proteins involved in a human reaction, the corresponding model organism reaction is computationally inferred and assembled with other inferred reactions into a model organism pathway. These pathways are accessible by choosing the model organism of interest from the drop-down menu on the Reactome website pathway browser (the default setting is, of course, *Homo sapiens*), and a species comparison tool allows users to visualize model organisms’ computationally inferred reaction sets in comparison to the curated human ones.

These pathways are computationally inferred, not manually curated like their human Reactome counterparts, and users should distinguish inferred events from experimentally supported human annotations when interpreting results.

### Tip 5: Provide the version of the data/tools used and acknowledge your sources

Reactome’s content is updated quarterly, and changes can affect pathway analysis results. To account for such changes, record the Reactome release date and version number alongside other parameters of your analysis. If you are using Reactome data via a third-party resource, check the version of the data that resource provides.

To detect possible effects of changes in Reactome on your analysis results, consider preserving your test results and successive versions of your data. Then re-do your test(s) and compare new results to previous ones to distinguish changes in your results that arise due to changes in successive versions of your data from those due to changes in successive versions of Reactome. Reactome’s history-tracking feature, named UpdateTracker, can help you identify whether relevant annotations were modified between releases.

When publishing work that uses Reactome, cite the relevant Reactome publications and acknowledge the version or tools used. Published citations of Reactome resources are essential to justify continued project funding. Current Reactome publications and citation information are available through the Reactome website. We suggest two papers that provide an up-to-date overview of the resource [15,16]. A comprehensive list of papers describing specific features of our data, annotation process and tools is available at https://reactome.org/community/publications.

### Tip 6: Get help to use Reactome effectively

Reactome offers a wide range of support resources. Start with a published tutorial [17] and on-line documentation, including an on-line data model glossary that defines all of the entity and event classes in the Reactome data model and the attributes of each, step-by-step guides, detailed explanations of analysis tools, and best practices for data interpretation.

If you prefer visual learning, explore Reactome’s on-line video tutorials (https://www.youtube.com/@Reactome) and recorded webinars, which walk through common workflows and advanced features.

For specific questions or technical challenges, reach out to the Reactome help desk (help@reactome.org). The support team can clarify analysis results, troubleshoot issues, and offer guidance tailored to your research needs.

Researchers can also request individual consultations for more in-depth assistance, especially when working with complex datasets or preparing figures for publication. Engaging with the Reactome team ensures you are using the resource effectively and staying aligned with current standards and updates.

### Tip 7: Contribute your expertise

Did your use of Reactome uncover areas of interest to you whose annotations are out of date or incomplete? Reactome is a community-curated resource, and researchers can contribute their expertise by reviewing existing pathway content or collaborating with Reactome curators to add or extend pathways. Use your expertise to improve and extend Reactome’s content by reviewing new and revised pathways, or by collaborating with us to fill annotation gaps. You will be credited with authorship or reviewership for all contributions. You can claim your Reactome contributions in ORCID using our ORCID claiming feature, and every pathway built in collaboration with outside experts is associated with a DOI that you can cite as a publication.

### Tip 8: Re-use our material

Reactome’s content and software are freely available for re-use on an open-source basis. A variety of data files and reports can be downloaded, and you can leverage Reactome’s open-access library of curated pathway diagrams and icons to create clear, consistent, publication-ready illustrations of proteins, complexes, and cellular events.

For example, a researcher developing a bioinformatics workflow can retrieve Reactome pathway data programmatically rather than manually downloading individual pathways. A machine-facing application programming interface (API) and related tools (reactome.org/dev) provide access to data, and can be used to integrate Reactome content into other on-line resources.

Reactome’s documentation provides information on available data, software, and reusable resources.

## Conclusions

Reactome is a comprehensive, freely accessible pathway knowledgebase that can significantly enhance biological interpretation when used effectively. By applying the practical strategies outlined in this article, from understanding pathway hierarchy and leveraging pathway enrichment tools to visualizing results thoughtfully and engaging with the Reactome community, researchers can better interpret their data in the context of biological pathways.

Importantly, Reactome is more than a static database; it is an actively curated, evolving resource supported by expert curators and a responsive scientific community. Making full use of its documentation, training materials, and user support services ensures that analyses are accurate, reproducible, and aligned with current pathway knowledge.

Whether you are conducting exploratory -omics analyses, preparing figures for publication, or designing follow-up experiments, thoughtful use of Reactome can strengthen the biological interpretation and impact of your research. By combining technical rigor with community engagement, researchers can maximize the value of this resource and contribute to more transparent and reproducible pathway-based science.

## Abbreviations

API: application programming interface

## References

[1] Vastrik I, D’Eustachio P, Schmidt E, Gopinath G, Croft D, de Bono B, et al. Reactome: a knowledge base of biologic pathways and processes. Genome Biol. 2007;8(3):R39. doi: 10.1186/gb-2007-8-3-r39 17367534 PMC1868929

[2] Kanehisa M, Goto S. KEGG: kyoto encyclopedia of genes and genomes. Nucleic Acids Res. 2000;28(1):27–30. doi: 10.1093/nar/28.1.27 10592173 PMC102409

[3] Karp PD, Riley M. Representations of metabolic knowledge. Proc Int Conf Intell Syst Mol Biol. 1993;1:207–15. 7584337

[4] Karp PD, Riley M, Paley SM, Pellegrini-Toole A. The MetaCyc database. Nucleic Acids Res. 2002;30(1):59–61. doi: 10.1093/nar/30.1.59 11752254 PMC99148

[5] Malik A, Arsalan M, Moreno C, Mosquera J, Félix E, Kizilören T, et al. ChEBI: re-engineered for a sustainable future. Nucleic Acids Research. 2026;54(D1):D1768–78.doi: 10.1093/nar/gkaf1271 41312627 PMC12807787

[6] Bansal P, Morgat A, Axelsen KB, Muthukrishnan V, Coudert E, Aimo L, et al. Rhea, the reaction knowledgebase in 2022. Nucleic Acids Res. 2022;50(D1):D693–700. doi: 10.1093/nar/gkab1016 34755880 PMC8728268

[7] UniProt Consortium. UniProt: the Universal Protein Knowledgebase in 2025. Nucleic Acids Res. 2025;53(D1):D609–D617. doi: 10.1093/nar/gkae1010 PMC11701636 39552041

[8] Gene Ontology Consortium. The gene ontology knowledgebase in 2026. Nucleic Acids Res. 2026;54(D1):D1779–92. 10.1093/nar/gkaf1292 PMC12807639 41413728

[9] Mohammadi H, Almodaresi F, Hogue GFJ, Wright A, Orlic-Milacic M, Li NT, et al. React-to-Me: a conversational interface for interactive exploration of the reactome pathway knowledgebase. BMC Biol. 2026. doi: 10.1186/s12915-026-02740-2

[10] Griss J, Viteri G, Sidiropoulos K, Nguyen V, Fabregat A, Hermjakob H. ReactomeGSA - efficient multi-omics comparative pathway analysis. Mol Cell Proteomics. 2020;19(12):2115–25. doi: 10.1074/mcp.TIR120.002155 32907876 PMC7710148

[11] Haw R, Loney F, Ong E, He Y, Wu G. Perform pathway enrichment analysis using ReactomeFIViz. Methods Mol Biol. 2020;2074:165–79. doi: 10.1007/978-1-4939-9873-9_13 31583638

[12] Beavers D, Brunson T, Sanati N, Matthews L, Haw R, Shorser S, et al. Illuminate the functions of dark proteins using the reactome-IDG web portal. Curr Protoc. 2023;3(7):e845. doi: 10.1002/cpz1.845 37467006 PMC10399304

[13] Wu G, Dawson E, Duong A, Haw R, Stein L. ReactomeFIViz: a Cytoscape app for pathway and network-based data analysis. F1000Res. 2014;3:146. 10.12688/f1000research.4431.1 PMC4184317 25309732

[14] Brunson T, Sanati N, Matthews L, Haw R, Beavers D, Shorser S, et al. Illuminating dark proteins using reactome pathways. bioRxiv. 2023. doi: 10.1101/2023.06.05.543335 PMC10399304 37467006

[15] Milacic M, Beavers D, Conley P, Gong C, Gillespie M, Griss J, et al. The reactome pathway knowledgebase 2024. Nucleic Acids Research. 2024;52(D1):D672–8. doi: 10.1093/nar/gkad1025 37941124 PMC10767911

[16] Ragueneau E, Gong C, Sinquin P, Sevilla C, Beavers D, Grentner A, et al. The reactome knowledgebase 2026. Nucleic Acids Research. 2026;54(D1):D673–81. doi: 10.1093/nar/gkaf1223 41251150 PMC12807730

[17] Rothfels K, Milacic M, Matthews L, Haw R, Sevilla C, Gillespie M, et al. Using the reactome database. Curr Protoc. 2023;3(4):e722. doi: 10.1002/cpz1.722 37053306 PMC11184634

